# How Cognitive Ability Shapes Personality Differentiation in Real Job Candidates: Insights from a Large-Scale Study

**DOI:** 10.3390/jintelligence12030034

**Published:** 2024-03-16

**Authors:** Alina N. Stamate, Pascale L. Denis, Geneviève Sauvé

**Affiliations:** 1 Department of Organization and Human Resources, School of Management Sciences, University of Quebec in Montreal, Montreal, QC H2X 3X2, Canada; denis.pascale@uqam.ca; 2 Department of Education and Pedagogy, University of Quebec in Montreal, Montreal, QC H2X 3R9, Canada; sauve.genevieve@uqam.ca

**Keywords:** personality, cognitive ability levels, job complexity levels, differentiation of personality, job candidates

## Abstract

The differentiation of personality by the cognitive ability hypothesis proposes that individuals with higher cognitive ability have more variability in their personality structure than those with lower cognitive ability. A large sample of actual job candidates (*n* = 14,462) who participated in an online proctored test session, providing socio-demographic information and completing cognitive ability, personality, and language proficiency assessments, was used to test this hypothesis. The total sample was divided into three equal groups (low, average, high) using percentiles as the cutoff point to investigate the effects of cognitive ability. An ANCOVA demonstrated the significant effect of cognitive ability on personality traits, controlling for language proficiency. Principal component analyses showed that the personality structure differed between the cognitive ability groups, with the high-cognitive-ability group having an additional personality component. Similarly, analyses across job complexity levels indicated more personality components for high-job-complexity positions. The implications, limitations, and future directions of this study are discussed.

## 1. Introduction

The study of individual differences in psychology has long focused on two fundamental domains: personality and cognitive ability. These domains hold immense significance across various areas of psychological research and practice, including organizational, developmental, and cultural psychology (Ellingsen 2016).

Personality is traditionally understood as a set of relatively stable characteristics that influence an individual’s preferences, motivations, and tendencies across different contexts (Carver and Scheier 2017). Understanding the nature of personality has led to the development of various models, each attempting to capture and describe the underlying dimensions that shape human behavior. One of the most prevalent personality models, the Five-Factor Model (FFM) (Costa and McCrae 1992), suggests that personality can be characterized by five broad factors: extraversion, agreeableness, conscientiousness, neuroticism, and openness to experience (McCrae and Costa 1987, 2008). These factors represent the most salient aspects of personality that are relatively stable across time and situations. However, some critics argue that this five-factor structure is limited in its ability to adequately capture the full spectrum of individual personality differences (e.g., Cattell 1993; Feher and Vernon 2021; Hough 1992; Strus and Cieciuch 2021). Indeed, alternative perspectives on personality structure have been proposed, with one to nine factors identified in previous studies attempting to represent the complexity of individual differences (e.g., Ashton and Lee 2007; Cheung et al. 2008; Howard and Van Zandt 2020; Lee and Ashton 2020; Nel et al. 2012; Rushton and Irwing 2008; Srivastava 2020). Despite these contradictory findings, the Five-Factor Model remains one of the most widely used structural frameworks in personality research and practice (Allik et al. 2017; Du et al. 2021). However, the ongoing debate surrounding personality structure fuels the need to explore alternative perspectives.

Turning our attention to cognitive ability, it refers to the mental capacities for processing information, reasoning, problem-solving, and learning, as well as adapting to, selecting, and shaping any environmental context, by using analytical, creative, and practical intelligence (Sternberg 1997). Researchers have proposed various models of cognitive ability to explain its structure, diversity, and individual differences across domains. These models of intelligence are based on different theoretical and methodological approaches, but they all include a general factor (g) or a positive manifold and several specific abilities (Feraco and Cona 2022). The role of g and the specific abilities varies depending on the model (Beaujean 2015). Some models assume that g directly influences the performance in all tasks (two-factor or oblique models), while others posit that specific factors mediate or moderate the relation between g and cognitive performance (hierarchical or bifactor models). Another perspective is that g is an epiphenomenon that emerges from a developmental association of originally independent specific abilities (Van Der Maas et al. 2006).

Both personality and cognitive ability can be measured using various instruments, such as self-report questionnaires, observer ratings, standardized psychometric tests, and performance tasks. Additionally, job complexity, which reflects the cognitive demands and challenges of different occupations, such as the amount of information processing, problem-solving, decision-making, learning, and creativity required for a job (Nye et al. 2022), could be used as an indirect measure of cognitive ability, as more complex jobs require higher cognitive capabilities (Schmidt and Hunter 2004).

The relationship between personality and cognitive ability has been a subject of interest and debate for over a century, yet its nature and direction remain unclear and disputed (Stanek and Ones 2023). One type of these relationships is the association between personality traits and cognitive ability, which indicates the degree to which they covary. Several studies investigated the associations between personality traits, especially those based on the Big Five Model (Costa and McCrae 1992), and cognitive ability, usually measured by intelligence tests or their subtests (Ackerman and Heggestad 1997; Rammstedt et al. 2023; Stanek and Ones 2023). Their results suggest that some personality traits are consistently and moderately related to some cognitive abilities, for example, openness to experience and fluid intelligence, emotional stability and crystallized intelligence, and conscientiousness and speed of processing. However, the associations vary depending on the level of specificity of the personality and cognitive ability constructs, as well as the characteristics of the samples and measures used.

Recent meta-analytic research offers intriguing insights into the potential relationships between cognitive ability and personality traits and facets (Anglim et al. 2022). This research suggests a complex and varied interplay, where the relationship between cognitive ability and the different personality dimensions is not straightforward and may vary across different facets and contexts. For openness to experience, there appears to be a notable association with higher cognitive ability, especially in areas like intellectual interests, creativity, and unconventionality. This might be due to the enhanced capacity of individuals with higher cognitive ability to process complex and abstract information, potentially fostering a mindset that is more receptive to new and unconventional ideas. However, this association is reportedly less pronounced in aspects of openness related to emotions and aesthetics, indicating that these facets might be influenced by a wider array of factors beyond cognitive ability (Anglim et al. 2022). When considering conscientiousness, this meta-analysis suggests a more subtle relationship with cognitive ability. While there might be some correlation with aspects like a lower preference for structured routines and a slightly higher sense of competence, this does not necessarily imply a direct relationship of cognitive ability with all facets of conscientiousness. In the case of extraversion, the overall correlation with cognitive ability remains unclear. Specific facets like sociability and assertiveness show different patterns of correlation, hinting that cognitive ability might be linked to certain expressions of extraversion, such as assertiveness, but not to others, like sociability. As for agreeableness, the findings indicate no direct correlation with cognitive ability. This suggests that traits associated with agreeableness, such as empathy and cooperation, are likely associated with a range of factors beyond cognitive ability. Finally, neuroticism and cognitive ability appear to be inversely correlated, with lower neuroticism associated with higher cognitive ability. This could suggest that higher cognitive ability provides better coping mechanisms and problem-solving skills, potentially reducing neurotic tendencies.

Another area of relationships is the causal influence between personality traits and cognitive ability, which implies that one domain affects the development or expression of the other.

Several theories have proposed possible explanations for the personality–cognitive ability relationship, such as Ackerman’s (1996) theory of adult intellectual development, and DeYoung’s (2015) cybernetic Big Five theory. These theories suggest that personality traits influence how people gain, use, and maintain cognitive ability through various processes such as interest, self-regulation, feedback-seeking, or learning strategies. Conversely, cognitive ability may influence how people perceive and display their personality traits through processes such as self-awareness.

In light of these two intersecting domains, the differentiation of personality by the cognitive ability hypothesis offers an interesting perspective on their relationship (Brand et al. 1994). This hypothesis posits that cognitive ability plays a significant role not only in cognitive processes but also in the organization of personality traits. It suggests that individuals with higher levels of cognitive ability possess greater cognitive resources and flexibility, enabling them to adaptively navigate and respond to various environmental demands. Consequently, they may demonstrate enhanced self-awareness, cognitive complexity, and a broader range of personality traits. The hypothesis predicts that at higher levels of cognitive ability, the variance among personality factors would increase, indicating a more differentiated personality structure. Conversely, at lower levels of cognitive ability, the variance among personality factors would decrease, indicating a less differentiated personality structure.

Despite its theoretical appeal, the differentiation of personality by the cognitive ability hypothesis has received limited empirical support. Previous studies using measures of the Big Five personality dimensions yielded mixed results regarding the effect of cognitive ability on personality structure. Some studies found support for the hypothesis, indicating greater variability in personality traits for individuals with higher intelligence (e.g., Escorial et al. 2019; Harris et al. 2006; Schermer et al. 2020a). However, other studies failed to find significant differences (e.g., De Fruyt et al. 2006; Mõttus et al. 2007; Waiyavutti et al. 2012). Inconsistencies were also observed in studies employing the 16PF (e.g., Austin et al. 2000; Murray et al. 2016; Schermer et al. 2020b) and the Personality Research Form (e.g., Harris et al. 2005; Schermer et al. 2020c).

The differentiation hypotheses in the study of cognitive ability suggest that specific cognitive factors become less influenced by general cognitive ability (g factor) across various levels of cognitive functioning (Feraco and Cona 2022). These hypotheses typically focus on how individual cognitive ability or personality traits are less overshadowed by overarching general factors as cognitive ability increases, rather than predicting the emergence of new factors. This understanding underpins much of the existing literature on cognitive ability differentiation, emphasizing the nuanced influence of general intelligence on specific cognitive domains or traits.

Building upon this foundation, our study seeks to extend the conversation from the realm of intelligence differentiation to the differentiation of personality by cognitive ability. While the structure and development of intelligence have been extensively studied, the ways in which the cognitive ability level might influence the structure and variability of personality traits remain less explored. Our research aims to fill this gap by examining the hypothesis that individuals with higher cognitive ability exhibit a more varied and complex personality structure than those with lower cognitive ability. This focus is not only relevant but necessary for advancing our understanding of the interplay between cognitive ability and personality structure, offering potential contributions to both theoretical frameworks and practical applications in fields such as personnel selection.

To advance our understanding of the complex relationship between intelligence and personality differentiation, it is important to consider individual factors that may influence this association, at least as covariates. One such factor is language proficiency, which involves the ability to effectively and appropriately use language in various contexts and situations (Verhoeven and Vermeer 2002). It encompasses not only the knowledge of linguistic components such as vocabulary, grammar, and pronunciation but also skills in using language for communication purposes (e.g., listening, speaking, reading, and writing) and requires background knowledge, critical thinking, metacognitive skills, as well as understanding and applying cultural nuances, beliefs, and practices (Verhoeven and Vermeer 2002). Previous research indicated that language proficiency is related to personality traits in various ways. For instance, language proficiency may influence how individuals perceive and express their personality traits in different languages or cultures (Chen and Bond 2010). It may also affect how individuals respond to personality items in self-report measures, as they may better understand the meaning and nuances of the items, allowing for finer distinctions between their own traits and those of others (Austin et al. 2006). Moreover, language proficiency may reflect the dimensionality and diversity of an individual’s thoughts, which, in turn, could impact their personality structure (Bowler et al. 2009), as they report it.

Considering the previous findings in the literature suggesting a potential relationship between cognitive ability and personality structure, the aim of this study was to examine the presence and diversity of personality dimensions among individuals with varying levels of cognitive ability. Job complexity was also utilized as an indirect measure of cognitive ability, reflecting the cognitive demands associated with different occupational roles. We anticipated that higher cognitive ability, as well as higher job complexity, might be associated with a more differentiated personality structure, characterized by an increased number of dimensions. Additionally, we acknowledged the potential influence of language proficiency on individuals’ personality structures, and therefore, it was included as a control variable in our analysis. By administering personality, cognitive ability, and language proficiency tests as part of the selection process, we sought to deepen our understanding of the intricate relationship between cognitive ability and personality traits. The large and diversified sample of actual job candidates allowed us to explore the relationship between cognitive ability and personality traits in a real-world context, enhancing the generalizability of our findings. This study aims to contribute to both theoretical advancements in the field of psychology and practical applications in personnel selection.

## 2. Method

### 2.1. Sample and Procedure

The data were collected in a real-life context of staff selection. The candidates were asked to participate in an online test session in a proctored environment. The confidential nature of the study was explained according to ethical standards. An informed consent was obtained from each participant prior to the testing session. The study was approved by an institutional research ethics board.

The participants were 14,462 candidates (32.8% women) for different positions (e.g., technician, professional, accountant) in a large Canadian service company. The average age of the respondents was 35.13 years (range: 18–74, SD = 10.36). Among the participants, 14.4% self-identified as belonging to a visible minority,^1^ 3.9% as belonging to an ethnic minority,^2^ 1.2% as belonging to an Indigenous community, and 0.7% as living with a disability.

### 2.2. Measures

A socio-demographic questionnaire documented the following variables: sex assigned at birth, age, minority self-identification, disability, last obtained diploma, and job categories sought. A general mental ability test was used as a measure of cognitive ability. Further, a personality inventory and a language proficiency test were also administered. All measures were administered in French.

#### 2.2.1. Cognitive Ability Test

Cognitive ability was measured using the Work Applied Cognitive Ability Test (WACAT, COMPMETRICA 2008a), a test that draws theoretical influence from Carroll’s three-stratum theory (Carroll 1993, 1996). The WACAT is designed to assess an individual’s ability to generalize their learning and knowledge to different situations, to think logically, and to perform mental operations of a verbal, spatial, and mathematical nature. Administered under timed conditions, the test comprises 50 items with incremental difficulty, providing a single total score indicative of an individual’s general intelligence level.

The WACAT exhibits strong validity in assessing cognitive ability, as evidenced by significant correlations with well-established cognitive tests, including the General Aptitude Test Battery (GATB; Droege 1984; Dvorak 1947) and the Wonderlic (Wonderlic 1983). Specifically, the correlation between WACAT and Wonderlic is substantial (*r* = 0.81), after correcting for variance restriction, while the correlation between WACAT and BGTA (overall result) is 0.71, similarly corrected. These strong correlations provide robust empirical support for the effectiveness of the WACAT in measuring the intended construct. Additionally, the WACAT has demonstrated consistent psychometric reliability (α = 0.80–0.86; *r*_test-retest_ = 0.87 **), establishing its utility as a valuable tool for assessing cognitive ability (COMPMETRICA 2008a). In the current study, an internal consistency analysis revealed that the WACAT exhibited a Cronbach’s alpha of 0.80 and a McDonald’s omega coefficient of 0.72, attesting to its satisfactory reliability. These psychometric properties, observed both previously and within our study, align with established standards, substantiating the WACAT’s credibility for assessing cognitive ability in the context of our research.

#### 2.2.2. Personality Inventory

Personality was measured using the Work Approach and Behavior Test (WABT, COMPMETRICA 2008b) that assesses 25 personality traits that can be categorized into the five major personality factors (α = 0.60 to 0.84; *r*_test-retest_ = 0.76 ** to 92 **) extraversion (E; min = 0; max = 60), agreeableness (A; min = 0; max = 48), conscientiousness (C; min = 0; max = 72), neuroticism (N; min = 0; max = 60), and openness to experience (O; min = 0; max = 60). In the context of the present study, the McDonald’s ω coefficients for personality traits ranged from 0.65 to 0.79, and the Cronbach’s α coefficients ranged from 0.65 to 0.76.

Additionally, the WABT incorporates three supplementary scales aimed at assessing social desirability, acquiescence, and response consistency (COMPMETRICA 2008b). It is noteworthy that the organization’s automated data administration and collection system performed the initial data cleansing related to these three indicators. 

It is essential to note that the WABT is designed to assess personality traits specifically in the context of work-related behaviors, acknowledging that these traits contextualized to the workplace may differ from context-free, generic personality constructs (Judge and Kammeyer-Mueller 2012; Wang and Bowling 2016). This contextualization in relation to the work environment is considered in the interpretation of our findings.

#### 2.2.3. Language Proficiency Test

A French language proficiency assessment, developed and validated by a linguistic evaluation service and endorsed by the Quebec Board of the French Language,^3^ was utilized by the Canadian service company participating in this study. The assessment quantitatively evaluated the candidates’ proficiency in various domains, i.e., grammatical, and customary spelling, syntax, vocabulary, and punctuation, through 100 multiple-choice items. The scores are presented as percentages, serving as indicators of the candidates’ proficiency levels. The candidates obtaining a score of 60% or higher were deemed to have met the criteria for progression to the subsequent stage of the selection process.

#### 2.2.4. Job Complexity

Different jobs require distinctive levels of cognitive ability depending on the complexity of the involved tasks (Denis et al. 2010; Hunter and Hunter 1984; Schmidt and Hunter 2004). In the collaborating organization, highly complex jobs require a university diploma, while less complex ones do not. For the purposes of this study, job complexity was operationalized through the required diploma level and categorized in two dimensions: low job complexity and high job complexity.

### 2.3. Statistical Analysis

Descriptive, mean differences, and principal component analyses with maximum likelihood extraction (Oblimin rotation and Kaiser standardization) were performed using SPSS version 28 software.

In this study, cognitive ability was categorized into low and high using percentiles, following the recommendation of Bowler et al. (2009). This approach was chosen to ensure a clear differentiation between groups and maintain balanced sample sizes, essential for a robust statistical analysis, and was aligned with group norms provided in the technical manual of the instrument. Focusing on the lower (below the 33rd percentile) and higher (above the 67th percentile) ends of the spectrum allowed for a targeted examination of the relationship between cognitive ability and personality structure, particularly in comparing low-cognitive-ability and high-cognitive-ability groups where significant differences were hypothesized.

Only the low-cognitive-ability (LCA) and high-cognitive-ability (HCA) groups were used for the analyses. While the average-cognitive-ability group (ACA) size was *n* = 4455 (32.1% women), the final sample size was *n* = 5228 (36.3% women) for the LCA group and *n* = 4779 (29.5% women) for the HCA group. A *t*-test for independent samples demonstrated a significant difference in mean cognitive ability levels between the LCA and the HCA groups (*t*_(9189.69)_ = −224.75, *p* < .001).

In line with our research objectives, we employed EFA as an analytical tool to delve into the intricate interplay between cognitive ability and personality dimensions. EFA was particularly suited for this study because it allowed for an open-ended exploration of the underlying factors without imposing predefined structures. By utilizing EFA with maximum likelihood extraction, Oblimin rotation, and Kaiser standardization, we were able to identify latent dimensions or factors that naturally emerged from our dataset. This approach is consistent with the exploratory nature of our research, as it enabled us to uncover the complexity and diversity of personality traits influenced by cognitive ability. Our focus on the number of factors generated through EFA aligns with the nuanced differentiation hypothesis that posits varying influences of general cognitive ability (g) on specific traits as cognitive ability levels differ.

For the job complexity groups, we classified the participants into low-job-complexity (LJC) and high-job-complexity (HJC) groups based on the highest level of education required for their current position. The expected education level for the LJC group was a non-university diploma (i.e., a diploma from a vocational or technical school or other non-university-level institution), while the expected education level for the HJC group was a university diploma (i.e., a diploma from a university-level institution). The collected data included the LJC group (*n* = 8720), the HJC group (*n* = 4576), and 1 166 missing responses on this variable.

A *t*-test for independent samples demonstrated a significant difference in mean cognitive ability levels between the LJC and the HJC groups (*t*_(10 436.87)_ = −61.17, *p* < .001).

Language proficiency was controlled for in the study to refine the analysis. This approach guaranteed that the results more accurately reflected the impact of cognitive ability on personality, devoid of the confounding effects of language skills. By accounting for the variation in language proficiency, which is shaped by diverse cultural and educational backgrounds (Lin 2019; Taras et al. 2021), the investigation into the primary relationship became clearer. This measure is in alignment with best research practices, thereby enhancing the validity of the findings through careful consideration of all pertinent variables.

## 3. Results

Before proceeding to the main analyses, we checked the hypothesis of the homogeneity of variances between the cognitive ability groups (LCA and HCA) using the Levene’s test of equality of variances. The results of this test are reported in Table 1. The Levene’s test was significant for all dimensions except for neuroticism, suggesting that most dimensions did not meet the assumption of homogeneity of variances. Therefore, we used the Welch’s t-test, which is a robust method of analysis of variance that does not depend on the hypothesis of homogeneity of variances, to compare the mean scores of the LCA and HCA groups on each dimension. The Welch method confirmed our hypothesis, showing that a different personality structure was found when comparing the LCA and HCA groups.

In terms of mean level differences, the HCA group scored significantly higher on agreeability and conscientiousness. In contrast, the LCA group scored significantly higher on extraversion and openness to experience. No mean differences were found in neuroticism levels between the HCA and LCA groups.

Correlational analyses between cognitive ability and language proficiency were performed. The result was statistically significant (*r* = 0.61 **). Also, a significant difference was observed for the level of language proficiency between the LCA and HCA groups (*t*_(9,725,678)_ = −76.80, *p* < .001).

Further, an ANCOVA was conducted to compare the cognitive ability groups (i.e., LCA vs. HCA) on each personality trait, while controlling for the effect of language proficiency. This analysis was chosen because it enabled a more precise comparison of the group means by controlling for the variance of the covariate.

For extraversion, the covariate language proficiency was significantly related to this personality dimension, F_(1, 10004)_ = 18.027, *p* < .001, η^2^*p* = 0.002. After adjusting for language proficiency, there was a significant effect of cognitive ability on extraversion, F_(1, 10004)_ = 277.382, *p* < .001, η^2^*p* = 0.027. Following Cohen’s (1988) guidelines for effect size interpretation, an η^2^*p* value of 0.027 suggested a moderate influence of cognitive ability on extraversion.

Language proficiency was significantly related to agreeability, F_(1, 10004)_ = 16.441, *p* < .001, η^2^*p* = 0.002. After adjusting for language proficiency, there was no significant effect of cognitive ability on agreeability, F_(1, 10004)_ = 0.210, *p* = .647, η^2^*p* < 0.01, suggesting that cognitive ability does not significantly influence this personality dimension.

For conscientiousness, language proficiency was significantly related to this dimension, F_(1, 10004)_ = 389.511, *p* < .001, η^2^*p* = 0.037. After adjusting for this covariate, there was a significant effect of cognitive ability on conscientiousness, F_(1, 10004)_ = 127.450, *p* < .001, η^2^*p* = 0.013, indicating a small but significant influence of cognitive ability (Cohen 1988).

Language proficiency was significantly related to neuroticism, F_(1, 10004)_ = 12.992, *p* < .001, η^2^*p* = 0.001. After adjusting for this covariate, there was a significant effect of cognitive ability on neuroticism, F_(1, 10004)_ = 23.892, *p* < .001, η^2^*p* = 0.002, showing a small influence of cognitive ability (Cohen 1988).

The covariate language proficiency was also significantly related to openness to experience, F_(1, 10004)_ = 5.052, *p* < .05, η^2^*p* < 0.01. After adjusting for language proficiency, there was a significant effect of cognitive ability on openness to experience, F_(1, 10004)_ = 53.256, *p* < .001, η^2^*p* = 0.005, indicating a small influence of cognitive ability (Cohen 1988).

Principal component analyses with maximum likelihood extraction (Oblimin rotation and Kaiser standardization) were used to examine the principal component results of the personality inventory for each cognitive ability group, separately. The results, which are detailed in Table 2, highlighted distinct patterns of personality components emerging across groups of varying cognitive ability and job complexities.

The Kaizer–Meyer–Olkin index (KMO) was observed to be higher for the LCA group than for the HCA group. The mean inter-scale correlation for the personality inventory factors for the HCA group was 0.19, which was lower than the mean inter-scale correlation of 0.23 for the LCA group. According to the eigenvalue criterion (Tabachnick and Fidell 2019), the principal component analysis revealed distinct patterns for each group (Kline 2016; Nunnally and Bernstein 1994).

For the LCA group, five principal components were identified, reflecting the classic dimensions of Costa and McCrae’s Five-Factor Model. These components, corresponding to openness, conscientiousness, extraversion, agreeableness, and neuroticism, accounted for 56.95% of the explained variance. The inventory items consistently loaded on these factors, supporting the theoretical model’s validity in this group.

In contrast, for the HCA group, six principal components were identified, with the additional sixth component explaining a total of 59.69% of the variance. This extra component, comprising subdimensions of dominance, persuasion, results orientation, work orientation, and action orientation, appeared to reflect traits related to motivation and goal orientation, particularly relevant in complex work contexts. Notably, these subdimensions do not align directly with an existing theoretical model but emerged as a distinct factor in this specific context, raising insightful questions about the personality structure in HCA populations and demanding work environments.

A second series of principal components analyses with maximum likelihood extraction (Oblimin rotation and Kaiser standardization) were used to verify if the personality structure was different across the levels of job complexity. The results are presented in Table 3. Further, a *t*-test for independent samples (*t*_(10,436.87)_ = −61.17, *p* < .001; Cohen’s *d* = −1.07) identified a significant difference between the groups.

These analyses suggested that there was a greater number of personality components for the HJC group. The results also showed an increased explained variance with a higher number of personality factors, as well as a lower KMO index value, for the HJC group.

## 4. Discussion

This study aimed to explore how cognitive ability affects the complexity of personality structure in a natural organizational setting of personnel selection. The results support our hypothesis suggesting that a more differentiated personality structure could be found at higher levels of cognitive ability.

### 4.1. Contextualization of Personality within the Work Environment

In this study, an instrument specifically tailored for assessing work-related behaviors was utilized to capture personality nuances within the employment context. This aligns with the debate on personality traits in context-specific scenarios (Bowling and Burns 2010; Schmit et al. 1995). This instrument considers unintentional factors affecting a test validity related to the frame of reference. Studies suggest that personality tests with a specific frame of reference demonstrate enhanced predictive validity (Pomerance and Converse 2014). Considering the assessment context is essential for precision and relevance in evaluating individuals.

### 4.2. Number of Distinct Dimensions of Personality Structure and Percentages of Variance Explained

Our study revealed a more differentiated personality structure in job applicants with higher cognitive ability. This pattern remained consistent when the cognitive ability level was measured both directly and indirectly by the job complexity level. In the LJC group, the traditional five-factor structure of personality was observed. However, in the HJC group, a sixth personality factor emerged.

The emergence of a sixth dimension in personality, which we labelled “personal drive”, provides a potentially novel perspective on the relationship between cognitive ability and personality structure. This dimension is interesting as it encompasses traits typically linked to various factors in the well-established Big Five Model. Our study suggests that in individuals with HCA, personality structure may be more differentiated, characterized by an increased number of dimensions. Specifically, traits that are usually associated with different dimensions of the Big Five Model—such as dominance and persuasion (commonly linked with extraversion), results and work orientation (typically connected to conscientiousness), and action orientation (often associated with neuroticism)—appeared to have converged in HCA individuals to form this additional dimension. The emergence of this sixth personality factor in the HCA and HJC groups allowed for more variance to be explained compared to the LCA and LJC groups. While this factor does not directly correspond to an existing theoretical model, it may reflect unique personality characteristics that become more prominent in complex work environments and among individuals with higher cognitive ability. This finding invites further reflection on how personality traits are structured and expressed across different contexts and populations.

One possible variable playing a role in differences in personality structure between individuals with varying cognitive ability may be language proficiency. The current study found a significant correlation between cognitive ability and language proficiency scores. The HCA individuals may have had more opportunities to develop and improve their language proficiency, which could have enabled them to have a more nuanced understanding of the personality items (Chen and Bond 2010; Dewaele and Furnham 2000). The differences in personality structure between the LCA and HCA groups in this study could therefore be partially explained by higher levels of language proficiency among individuals with HCA.

Moreover, it could be that the HCA individuals exhibited a more differentiated personality structure because of their cognitive resources and adaptability. HCA individuals may show enhanced cognitive ability, capacity for cognitive complexity (i.e., perceiving multiple aspects of situations or individuals), and self-awareness (i.e., reflection on one’s thoughts, feelings, and behaviors), potentially enabling a clearer differentiation of their personality traits (Carver 2012). The idea of enhanced cognitive ability possibly leading to a broader range of personality traits and stronger trait relationships was also proposed by others (Bieri 1966; Demetriou et al. 2020).

Another possible explanation for the difference in personality structure is the role of social desirability, especially in high-stakes contexts like personnel selection. People tend to present themselves in a socially favorable way (Goffman 1959), which is more pronounced when their responses face scrutiny and judgment (Krumpal 2013). This social desirability bias could have had a significant impact on the HCA participants in our study as they completed the workplace-contextualized personality inventory. This contextualization may have influenced how the participants reported their personality because they perceived that traits like dominance, persuasion, results orientation, and action orientation could be valued in professional settings.

The possibility that the relationship between cognitive ability and personality facets undergoes changes at different levels of cognitive functioning is another aspect to consider. As mentioned earlier, the differentiation hypotheses primarily focus on the idea that specific cognitive factors become less influenced by general cognitive ability (g) as cognitive functioning levels increase (Feraco and Cona 2022). This concept is often applied to cognitive domains or personality traits, suggesting a reduced influence of overarching general factors. The emergence of the “personal drive” personality dimension in our study at higher levels of cognitive ability raises the question of whether, at these elevated cognitive ability levels, the various facets of personality could become less overshadowed by overarching general factors. This possibility adds to traditional models of cognitive ability differentiation and highlights the need for further exploration into the intricate interplay between cognitive ability and the multifaceted nature of personality.

Furthermore, the distinction in the percentage of variance explained by the principal components, as revealed by our EFA, offers a quantitative foundation to these qualitative observations. The fact that 59.69% of the variance could be accounted for in the HCA group by six principal components, compared to 56.95% accounted for by five components in the LCA group, numerically underscores the more differentiated personality structure associated with higher cognitive ability. Similarly, the variance explained in the HJC group (58.65%) by six components, in contrast to that in the LJC group (57.24%) explained by five components, is aligned with the complexity of personality structures in contexts demanding higher cognitive engagement.

These percentages reinforce the emergence of the “personal drive” dimension and highlight the nuanced interplay between cognitive ability and personality structures. They suggest that the breadth and complexity of an individual’s personality may expand in response to enhanced cognitive capabilities and environmental demands, such as those found in more complex job settings. This observation aligns with the differentiation hypothesis (Brand et al. 1994), which posits that higher levels of cognitive functioning are associated with a more nuanced and differentiated personality structure.

### 4.3. Differences between the Groups on the Personality Dimensions

After conducting an ANCOVA and accounting for language proficiency as a covariate, we found that cognitive ability still significantly influenced most personality traits. Of note, the impact of cognitive ability on agreeableness became non-significant when language proficiency was considered. This suggests that while language proficiency influences some personality traits, cognitive ability independently affects several personality dimensions and could rather have a potential mediating role on certain traits. These findings align with Anglim et al.’s (2022) meta-analysis, which also found no direct correlation between cognitive aptitude and agreeableness.

Two unexpected mean differences should be noted. First, the HCA group had significantly lower scores for the openness to experience factor compared to the LCA group. Previous research showed a positive correlation between this personality dimension and cognitive ability scores (*r* = 0.30; Ackerman and Heggestad 1997; DeYoung 2011; DeYoung et al. 2014). However, our study’s personality inventory specifically measured the acceptance of unconventional ideas within openness to experience. A possible social desirability bias should be considered, given our study’s personnel selection context (Schermer et al. 2020b; Williams 2002). Further investigation is needed to explore this finding, as our instruments did not assess the level of social desirability.

Another unexpected finding was the lack of a significant mean difference between the LCA and HCA groups for neuroticism. Previous studies reported that this personality dimension showed a weak negative correlation with cognitive ability scores (*r* = −0.15; Ackerman and Heggestad 1997), possibly due to test anxiety (Denis et al. 2022). In the current study, the hiring organization had put some measures in place to reduce this type of anxiety. For example, the cognitive ability test was timed, but the time granted was sufficient for the test to be completed by all. Thus, time pressure was probably not an influencing factor during the test session, and this could partially explain our findings. Future research could use more comprehensive measures of neuroticism to explore its relationship with cognitive ability.

### 4.4. Contributions

This study contributes to the understanding of the differentiation of personality by cognitive ability in a real-life personnel selection context. It stands out by using a comprehensive personality inventory and examining its relationship with cognitive ability levels with a sample of diverse candidates in terms of type and job position targeted. The findings provide empirical evidence that individuals with HCA exhibit a more differentiated personality structure, highlighting the importance of considering both cognitive and personality factors in personnel selection and job performance prediction.

The emergence of a sixth personality factor, “personal drive”, at the HCA and HJC level adds to our explorative endeavors on the multidimensional nature of personality. This factor encompasses traits related to dominance, persuasion, results orientation, work orientation, and action orientation, reflecting the motivational and goal-oriented aspects of personality that become more salient in complex work settings. By identifying this additional factor, organizations could gain insights into the specific personality traits that might be most relevant for success in high-complexity jobs.

This study adds to the current literature by measuring cognitive ability both directly and indirectly. Until now, the differentiation of personality by the cognitive ability hypothesis was under-analyzed in high-stakes contexts, such as job selection processes (DuVernet et al. 2014; McLarnon and Carswell 2013; Schermer et al. 2020b).

Based on our findings, organizations should consider incorporating cognitive ability measures alongside personality assessments in personnel selection processes. This holistic approach can enhance the job–person fit, benefiting performance and productivity. Additionally, accounting for language proficiency in personality tests could ensure more accurate trait measurement, especially for individuals with varying cognitive ability.

### 4.5. Limitations and Future Directions

Several limitations should be acknowledged.

First, the sample consisted of job candidates from a specific hiring organization, which may limit the generalizability of the findings to other populations. Yet, some generalization can still be possible, given that our sample consisted of candidates for diverse jobs and fields of work. Replication is warranted.

Second, our analyses were limited to personality factors and used a cross-sectional design due to logistic limitations. Austin et al. (2000) suggested that individuals with higher cognitive ability levels should have higher reliability values in their personality inventory scores. Potential differences in instrument reliability for individuals with different levels of cognitive ability is an area requiring further exploring (Escorial et al. 2019; Navarro-González et al. 2018).

Third, while we controlled for language proficiency’s influence on the relationship between cognitive aptitude and personality structure (Pacifico et al. 2023), future research could explore its potential mediating roles, as suggested by others (Bowler et al. 2009; Chen and Bond 2010; Chen et al. 2022; Rosselli et al. 2017; Veltkamp et al. 2013; Vygotsky 1934, 1978; Weinert 2022). It is important to note, however, that while general cognitive ability includes a language component, our assessment tool did not allow for separating this component from the overall g factor, posing a limitation in our study’s ability to isolate the specific effects of language proficiency on personality structure. Future research could benefit from more directly measuring the language proficiency’s role to deepen the understanding of its influence within the cognitive framework. Moreover, future research could benefit from a more detailed examination of the psychometric properties of the French language proficiency assessment, particularly given the limitation that detailed scientific validation results of this test were not accessible for this study. Specifically validating the instrument for the study’s context would further elucidate its reliability and applicability in accurately measuring candidates’ language skills.

Fourth, the classification of job complexity in this study was based on the participants’ highest level of education required for their current positions. While this approach aimed to create distinct categories for practicality, it may not fully capture the multidimensional nature of job complexity. Future research could add factors like task nature, decision-making autonomy, skill variety, and dynamic work environments to their operationalization.

Fifth, the observed effects, though statistically significant, were relatively modest in size (Cohen 1988). This suggests that cognitive ability is likely one of many factors influencing personality structure.

Sixth, to investigate the role of cognitive ability on personality dimensionality, our study initially categorized the sample into three comparable groups, following the approach of Bowler et al. (2009). While this method simplified the analysis, it introduced the risk of artificial dichotomization. We acknowledge the potential benefits of employing the moderated factor model or local structural equation modeling, as recommended by Molenaar et al. (2010) and Robitzsch (2023), which evaluates if personality factor loadings vary across levels of cognitive ability. This approach, which models cognitive ability on a continuous scale, could complement traditional categorization methods by providing additional insights into how factor loadings vary with cognitive ability levels. Despite its advantages, this method was not considered in our current research due to data limitations and its aim being beyond the scope of our study. Nevertheless, we recommend comparing our findings with those of such moderated factor modeling approach in future research, especially since its application at the item level, as hinted by Murray et al. (2016), could provide deeper insights into the nuances of personality differentiation in relation to cognitive ability. This recommendation underscores a promising direction for advancing the field.

A final limitation of our study is how response biases, such as acquiescence and socially desirable responding, influenced the personality assessments. While we integrated measures within the Work Approach and Behavior Test (WABT) to address these biases, it is essential to acknowledge their potential impact on the assessment outcomes (Kreitchmann et al. 2019). The organization’s automated data administration and collection system performed the initial data cleansing related to these biases, thereby enhancing the reliability and validity of our findings. However, it is important to recognize that even with automated procedures, nuances in word meanings and situational demands may still shape response patterns (Danner et al. 2015; Toomela 2003; Ziegler et al. 2009). Future studies should further explore these intertwined influences using multi-source and multi-method approaches for a more comprehensive personality assessment, while also examining cognitive abilities and considering the specifics of the assessed population.

Analyses like structural equation modeling and latent profile analyses could also provide deeper insights, although they were not feasible in our study.

## 5. Conclusions

In conclusion, this study tested the hypothesis that personality structure is more differentiated at higher levels of cognitive ability in a real-life personnel selection context. We found evidence to support this hypothesis, regardless of whether cognitive ability was measured directly by a general mental ability test or indirectly by the job complexity level. These findings have implications for personnel selection, job performance prediction, and understanding the interplay between cognitive and personality factors in the workplace.

Such knowledge can have practical implications in fields like education, personnel selection, and organizational psychology, where tailored interventions and strategies can be developed to maximize individual potential and improve overall outcomes. Using a fixed personality structure for all individuals or jobs may not capture all the relevant dimensions of personality that vary across different levels of cognitive ability or job complexity. Therefore, practitioners who use personality instruments in selection contexts should be aware of the potential limitations and biases that may arise from using a one-size-fits-all approach. Alternatively, they could use more adaptive and flexible personality measures that can adjust to individual differences in personality structure, such as CAT (computerized adaptive testing)- or IRT (item response theory)-based instruments (DuVernet et al. 2014; McLarnon and Carswell 2013).

## Figures and Tables

**Table 1:** Means (M), standard deviations (SD), and Levene’s F-test of homogeneity of variance values for the lower and upper cognitive ability groups, for the cognitive ability test and personality inventory.

Dimension	LCA Group M (SD)	HCA Group M (SD)	Levene’s *F* Test of Variance	Welch’s *t-*Value (*df*)	Cohen’s *d*	95% CI Cohen’s *d*
CA	25.30 (3.98)	40.43 (2.67)	701.99 ***	−224.75 (9189.69) ***	−4.43	[−4.49, −4.36]
E	87.89 (5.87)	84.81 (5.99)	8.55 **	24.08 (9869.08) ***	0.52	[0.40, 0.64]
A	67.87 (9.32)	68.38 (8.88)	18.61 ***	−2.82 (9988.18) **	−0.06	[−0.23, 0.12]
C	95.35 (8.36)	99.10 (9.62)	107.46 ***	−20.71 (9511.91) ***	−0.42	[−0.59, −0.24]
N	84.51 (8.62)	84.21 (8.84)	5.10 *	1.71 (9873.68)	0.03	[−0.14, 0.21]
O	83.22 (9.08)	81.87 (9.87)	34.83 ***	7.14 (9717.25) ***	0.14	[−0.04, 0.33]

Note. *n*_LCA_ = 5228; *n*_HCA_ = 4779; *** *p* < .001; ** *p* < .01; * *p* < .05, two-tailed; CA = cognitive ability; E = extraversion; A = agreeability; C = conscientiousness; N = neuroticism; O = openness to experience; according to Cohen (1988), *d* = 0.2 (weak effect), *d* = 0.5 (moderate effect), and *d* = 0.8 (strong effect).

**Table 2:** Principal component analysis for the lower and upper cognitive ability groups for the personality inventory.

Subsample	KMO	χ^2^_(300)_	Explained Variance	Number of Principal Components
*LCA*	0.833	45,929.19 ***	56.95%	5
*HCA*	0.818	40,522.27 ***	59.69%	6

Note. *n*_LCA_ = 5228; *n*_HCA_ = 4779. *** *p* < .001, two-tailed.

**Table 3:** Principal component analysis for the low- and high-job-complexity groups for the personality inventory.

Subsample	KMO	χ^2^_(300)_	Explained Variance	Number of Principal Components
*LJC*	0.831	78,514.21 ***	57.24%	5
*HJC*	0.810	36,669.18 ***	58.65%	6

Note. *n*_LJC_ = 8 720; *n*_HJC_ = 4 576; *** *p* < .001, two-tailed.

## Data Availability

The data presented in this study are available on request from the corresponding author due to an agreement of confidentiality with the data-providing company that restricts the public sharing of these data.

## Notes

1 For the purposes of this study, belonging to a visible minority is based on the person’s “race or the colour of their skin” (Act Respecting Equal Access to Employment in Public Bodies 2000).

2 For the purposes of this study, people belonging to an ethnic minority refers to “persons whose mother tongue is neither French nor English and who belong to a group other than the Aboriginal people group or the visible minority group” (Act Respecting Equal Access to Employment in Public Bodies 2000).

3 https://www.oqlf.gouv.qc.ca/ (accessed on 24 January 2024).
